# Validation of Novel Relative Orientation and Inertial Sensor-to-Segment Alignment Algorithms for Estimating 3D Hip Joint Angles

**DOI:** 10.3390/s19235143

**Published:** 2019-11-24

**Authors:** Lukas Adamowicz, Reed D. Gurchiek, Jonathan Ferri, Anna T. Ursiny, Niccolo Fiorentino, Ryan S. McGinnis

**Affiliations:** 1 M-Sense Research Group, University of Vermont, Burlington, VT 05405, USA; lukas.adamowicz95@gmail.com (L.A.); reed.gurchiek@uvm.edu (R.D.G.); Jonathan.Ferri@uvm.edu (J.F.); Anna.Ursiny@uvm.edu (A.T.U.); 2 Department of Mechanical Engineering, University of Vermont, Burlington, VT 05405, USA; Niccolo.Fiorentino@uvm.edu

**Keywords:** 3D joint kinematics, joint angles, inertial measurement units, inertial sensors, wearable sensors, IMU, motion tracking, functional rotation axes

## Abstract

Wearable sensor-based algorithms for estimating joint angles have seen great improvements in recent years. While the knee joint has garnered most of the attention in this area, algorithms for estimating hip joint angles are less available. Herein, we propose and validate a novel algorithm for this purpose with innovations in sensor-to-sensor orientation and sensor-to-segment alignment. The proposed approach is robust to sensor placement and does not require specific calibration motions. The accuracy of the proposed approach is established relative to optical motion capture and compared to existing methods for estimating relative orientation, hip joint angles, and range of motion (ROM) during a task designed to exercise the full hip range of motion (ROM) and fast walking using root mean square error (RMSE) and regression analysis. The RMSE of the proposed approach was less than that for existing methods when estimating sensor orientation ($12.32^{{}^{\circ}}$ and $11.82^{{}^{\circ}}$ vs. $24.61^{{}^{\circ}}$ and $23.76^{{}^{\circ}}$) and flexion/extension joint angles ($7.88^{{}^{\circ}}$ and $8.62^{{}^{\circ}}$ vs. $14.14^{{}^{\circ}}$ and $15.64^{{}^{\circ}}$). Also, ROM estimation error was less than $2.2^{{}^{\circ}}$ during the walking trial using the proposed method. These results suggest the proposed approach presents an improvement to existing methods and provides a promising technique for remote monitoring of hip joint angles.

## 1. Introduction

Joint angle estimates are emerging as important metrics for the analysis of human health and performance. They may soon play key roles in the identification and treatment of a variety of conditions impacting mobility such as osteoarthritis (OA) [1], multiple sclerosis (MS) [2], Parkinson’s disease (PD) [3], stroke [4], and rehabilitation from joint injury [5,6]. Hip range of motion, in particular, has emerged as an important indicator of lower-limb motor impairment [7,8], and differentiates healthy and obese children [9]. However, advancement in the use of joint angles for the analysis of human health and performance is largely inhibited by limitations of the current measurement modalities employed in both clinical and research contexts.

The accepted standard for joint angle estimation is optical motion capture (OMC). OMC works by using infra-red (IR) cameras to locate IR-reflective markers in 3D space. Markers are placed on specific anatomical landmarks (e.g., anterior-superior iliac spines, femoral epicondyles, etc.) which allow the reconstruction of segment frames. The relative orientation of adjacent frames is then used to compute the joint angles of interest [10]. OMC suffers from several limitations including a finite capture volume, high cost, setup complexity, and the need for little or tight-fitting clothing to prevent marker occlusion. These limitations prevent the broad use of OMC outside of research institutions and limit the types of activities that can be observed. Nevertheless, OMC remains the gold standard research method for capturing human movement, and especially joint angles. However, shortcomings of OMC have motivated the development of alternative methods for measuring joint kinematics. With the recent advances in micro-electromechanical systems (MEMS), inertial measurement units (IMUs) are being utilized with increasing frequency in biomechanics to estimate joint angles [11,12,13,14,15,16,17,18,19,20,21,22,23,24,25,26,27,28].

IMUs are comprised of an accelerometer and an angular rate gyroscope, which measure linear acceleration and angular velocity respectively. Some IMUs also contain a magnetometer, which measures the local magnetic field, and are referred to as magnetic and inertial measurement units (MIMUs). Accelerometer, gyroscope, and potentially magnetometer data from these sensors are often fused together to estimate the relative orientation of the IMU (e.g., [29,30]) and, when deployed to adjacent body segments, joint angles. Critical to most joint angle estimation algorithms are the estimation of the sensor’s orientation relative to a relevant reference frame [11,31], mapping sensor coordinate systems to their respective segment reference frames [11,14,16,31], and compensating for drift [11,32] (the accumulation of error in the estimate over time due to bias and approximations in integration). Previous studies have sought to address each of these components, but room for improvement remains in each area.

Previous approaches for sensor orientation estimation have shown good results relative to OMC, though it is sometimes unclear how well these methods generalize to situations requiring extended measurement of dynamic activities or experiencing significant magnetic interference. One popular method used gradient descent optimization, and achieved root mean square error (RMSE) values between $0.581^{{}^{\circ}}$ and $1.110^{{}^{\circ}}$ for Euler angles deconstructed from the direction cosine matrix, when compared to motion capture [33] for foot tracking during walking. A Kalman filter (KF) approach tracked hand waving, arm exercises, and walking with RMSE values all less than $1.63^{{}^{\circ}}$ for roll and pitch angles when compared to a tactical grade IMU [34]. However, previous methods have not addressed or exploited the constraints imposed by adjacent body segments, which likely would allow for improved error correction over a single sensor approach [16].

Previous approaches for joint angle estimation with wearable inertial sensors have focused primarily on the knee (e.g., see [14,15,16]). Examples of hip angle estimation are limited, and those examples that do exist use proprietary algorithms, limit their analysis to only a subset of the data collected, and/or do not consider data recorded during walking. One study examining the performance of commercial algorithms for estimating hip angles reported RMSE values less than $7.5^{{}^{\circ}}$ during manual material handling tasks [31], while another reported RMSE values of $9.6^{{}^{\circ}}$ and $27.6^{{}^{\circ}}$ during walking and running respectively [35]. The challenge with these proprietary algorithms is that information about the specific algorithm and assumptions used in its derivation are often limited, and purchase of the associated sensor system is required for replication and deployment. One study, which provides open-source algorithms, found mean errors less than $10.6^{{}^{\circ}}$ during treadmill alpine skiing [11]. The approach required a comprehensive set of calibration motions and the analysis was not extended to walking data. Finally, a recent study found mean error magnitudes of less than $7.2^{{}^{\circ}}$ during walking, jumping jacks, squats, and jump turns [32]. However, 60% of hip internal-external rotation (IER) trials were excluded from analysis because they contained significant drift, limiting confidence in the deployment of this approach. These results highlight the emerging nature of solutions for estimating hip joint angles. Namely, very few methods validate during clinically relevant tasks such as gait, employ approaches that rely only on MIMU data, or are open source. Additionally, comparisons to clinically relevant metrics such as range of motion (ROM) [1,2,3], and the associated discussions are lacking from existing literature making it unclear if the reported algorithm performance is sufficient for resolving the quantities that motivate the work.

Building on this previous work, the objectives of this study were to carefully validate novel sensor-to-sensor relative orientation and sensor-to-segment alignment algorithms by assessing performance in the estimation of relative orientations and hip joint angles in human subjects. To provide context for these results, their performance was also compared to other methods previously described in the literature [11,13]. Finally, to examine the potential clinical utility, the computed joint ROMs are also compared to those from OMC.

## 2. Methods

### 2.1. Measurement Protocol

Twenty subjects (N = 10 male, N = 10 female, 22.9±5.4 years old; Inclusion: able to perform daily activities without difficulty; exclusion: diagnosis of a balance or mobility impairment, inability to complete the in-lab activities of daily living without assistance, opioid-dependent) participated in the study. The study was carried out following the rules of the Declaration of Helsinki of 1975, and all study activities were approved by the University of Vermont Institutional Review Board (CHRBSS 18-0518, approved 24 May 2018) and subjects gave written informed consent prior to participation.

**Figure 1:**
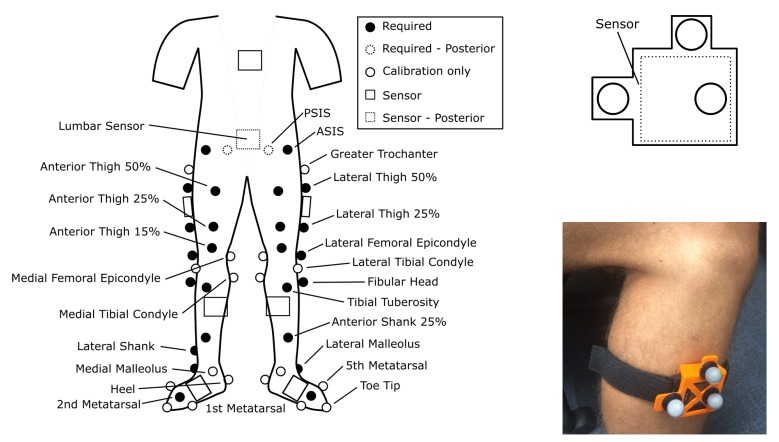
Marker set used, including calibration only markers, and close up views of the sensor clip used to create marker clusters (schematic—top right, example of clip attached to a leg—bottom right). Dotted lines indicate the sensor or marker is located on the posterior side.

Subjects were instrumented with wearable MIMU sensors and reflective markers for OMC, as in Figure 1. Two calibration trials were performed, a static standing and a star calibration motion (‘StarArc’ in [36]. Five combined movements were used herein instead of seven). Following the calibration trials, calibration only markers (trochanters, medial femoral epicondyles, lateral tibial condyles, medial tibial condyles) were removed before subjects completed a series of activities including standard functional assessments (e.g., standing-sitting transitions) as well as simulated activities of daily living (e.g., walking, lying down). Each subject performed each task once.

Herein we consider data sampled during trials when subjects completed the star calibration and walked on the treadmill for one minute (‘walking’ trial). The star calibration was included because it exercised the hip in all three axes of rotation, while the treadmill walking was included as a common daily activity that is also used in clinical assessments. Over all the subjects, the star calibration task had a duration of 56.41±9.99 s (min: 38.44 s, max: 74.55 s). Treadmill walking speed was self-selected fast (resulting speed: 4.89±0.68 kph, min: 3.50 kph, max: 6.40 kph, kph: kilometers per hour), with the speed chosen to validate on a more dynamic motion than walking slower would represent.

### 2.2. Optical Motion Capture

Reflective markers were tracked at a rate of 100 Hz with a 19-camera optical motion capture system (VICON Motion Systems) covering two overlapping capture volumes (a force plate walkway and treadmill). Subjects had markers placed on body segments and segment anatomical landmarks, following the locations suggested in [37,38,39]. Rigid clusters of three markers (see Figure 1) were also attached to each inertial sensor.

Reference rotations from a thigh sensor to the pelvis sensor were computed as the ground truth. This required both the cluster to global orientation, and the sensor to cluster orientation. To find the time-invariant sensor to cluster orientation, the cluster angular velocity was computed per

(1) ωC=RTCGdRCGdt,

where ω is the angular velocity, *C* indicates the cluster frame and *G* the global/world frame, and RBA is the rotation matrix from *B* to *A*. The OMC global frame is aligned with the capture volume used, and is consistent per subject for all OMC computations. RCG was computed using the cluster marker positions. The cluster angular velocity was then compared to the measured sensor angular velocity to obtain RSC (*S* is the sensor reference frame) using the singular value decomposition (SVD) [40]. The OMC-based sensor-to-sensor rotation can then be found per

(2) R21=RT1C1RTC1GRC2GR2C2,

where 1 and 2 indicate a sensor, for example, 1 and 2 could indicate the lumbar and a thigh sensor, respectively.

Hip joint angles were computed from OMC data following an established approach. First, functional joint centers were computed using a least squares geometric fit method [41] with an additional bias correction optimization to compensate for any soft-tissue artefact (STA) in marker trajectories [42]. Segment anatomical frames were defined per ISB standards [37,38] during the static calibration trial, and constant cluster-to-anatomical frame rotations were computed. Hip joint angles were computed as suggested in the ISB standards [37] with angle range corrections in the flexion-extension (FE) and IER directions [43]. Specifically, hip joint axes ($e_{1}$, $e_{2}$, $e_{3}$) were defined such that $e_{1}$ is aligned with the *Z*-axis of the pelvis (*X*, *Y*, *Z* are the pelvis axes), $e_{3}$ is aligned with the *y*-axis of the thigh (*x*, *y*, *z* are the thigh axes), and $e_{2}$ is perpendicular to each ($e_{2}=e_{3}\times e_{1}$). Hip joint angles are then defined per

(3) $$\begin{array}{cc} \alpha & =arctan2\left(\eta_{\alpha }||X\times e_{2}{\left|\right|}_{2},\;X\cdot e_{2}\right)\\ \eta_{\alpha } & =\frac{(X\times e_{2})\cdot Z}{\left|(X\times e_{2})\cdot Z)\right|} \end{array}$$

(4) $$\begin{array}{cc} \gamma & =arctan2\left(\eta_{\gamma }||x\times e_{2}{\left|\right|}_{2},\;x\cdot e_{2}\right)\\ \eta_{\gamma } & =\left\{\begin{array}{c} right\;\;\left(\left(x\times e_{2}\right)\cdot -y\right){\left[|(x\times e_{2})\cdot -y|\right]}^{-1}\\ left\;\;\;\;\left(\left(x\times e_{2}\right)\cdot y\right){\left[|(x\times e_{2})\cdot y|\right]}^{-1} \end{array}\right. \end{array}$$

(5) $$\begin{array}{cc} \beta & =\left\{\begin{array}{c} right\;\;-\frac{\pi }{2}+arccos\left(e_{1}\cdot e_{3}\right)\\ left\;\;\;\;\;\;\;\;\frac{pi}{2}-arccos\left(e_{1}\cdot e_{3}\right) \end{array}\right. \end{array}$$

where α is the FE angle, γ is the IER angle, β is the ad/abduction (AA) angle, $\left|\right|\cdot {\left|\right|}_{2}$ is the vector 2-norm, and η is a correction that ensures the sign conventions are maintained. The OMC-based hip joint angles and sensor-to-sensor rotations serve as ground truth values for comparing the existing and novel MIMU-based algorithms described next.

### 2.3. Wearable Magnetic and Inertial Sensors

Eight MIMU sensors (Opal v2, APDM, Inc. ‘Opal’) were each seated in plastic clips and attached to feet, shanks, thighs, the lumbar, and sternum in manufacturer-suggested locations (see Figure 1) via double-sided adhesive tape and velcro straps to prevent slipping against the skin (see Figure 1). However, the algorithm presented herein requires a minimum of three permanent and one temporary (only used initially for joint center estimation) sensor for bilateral estimation, whereas only two permanent and one temporary sensor would be required for unilateral joint angle estimation. Herein, only the lumbar and thigh sensors are considered for joint angle estimation, and the shank sensors utilized only for joint center location estimation.

Data from all Opals were time-synchronized with each other, and were synchronized with the OMC system via an electronic trigger. Acceleration, angular velocity, and magnetic field were recorded from each sensor at a sampling frequency of 128 Hz.

### 2.4. Existing Methods for Reference

#### 2.4.1. Sensor-to-Sensor Rotation

Performance of the current state of the art algorithms for estimating sensor orientations on these data was established using the proprietary orientation estimation provided by the Opal sensor manufacturer APDM (‘APDM’ orientation method). As the Opal orientation output is per sensor relative to a world frame (defined by gravity and magnetic north), the sensor-to-sensor orientation was defined as

(6) R21=RG1R2G.

#### 2.4.2. Hip Angles

Performance of current state of the art hip angle estimation algorithms was established using one of the few, and potentially only, open-source algorithms available (functional calibration—strap down integration (FC-STI)) [11,12,13] (MATLAB source code was ported to Python and validated against provided sample data. Original MATLAB code available: https://codeocean.com/capsule/1305245/tree/v1). This method requires a series of functional calibration activities (left and right hip ad/abduction, squatting, trunk rotations, standing) that are used to determine anatomical rotation axes. The orientation of these axes over time is computed using strap-down integration with joint kinematic constraints used to correct for drift error. Best performance is achieved when data from sensors deployed to all lower-body segments (shanks, thighs, pelvis) and the sternum are considered. This approach provides the pelvis and thigh anatomical frames necessary for computing hip joint angles as per Equations (3)–(5).

### 2.5. Novel MIMU Methods

As part of this work, two novel methods were developed. First, we propose a novel KF-based method for estimating sensor-to-sensor relative orientations (SSRO), which allows data in the thigh-sensor local frame to be transformed into the pelvis-sensor local frame. Next, a novel method for obtaining the sensor-to-segment alignments was developed, utilizing computed joint center locations to form the anatomical functional rotation axes (the ‘proposed’ method). These axes correspond, as closely as possible, to those used by OMC for computing hip joint angles. Finally, joint angles were computed as the angle between specific anatomical axes, as in the OMC method.

#### 2.5.1. Data Preprocessing

Measured angular velocity, acceleration, and magnetometer readings were low-pass zero-phase filtered with a 15 Hz cutoff frequency. Angular acceleration was calculated from angular velocity using a second order approach per

(7) $$\begin{array}{cc} {\dot{\omega }}_{i}^{k} & =\frac{\omega_{i}^{k+1}-\omega_{i}^{k-1}}{2\Delta t}, \end{array}$$

where the overhead dot indicates the time derivative, as in ${\dot{\omega }}_{i}^{k}$ is the angular acceleration of the *i*-th sensor at time point *k*, and Δt is the time difference between adjacent samples. After calculation, angular acceleration was low-pass zero-phase filtered with a cutoff frequency of 12 Hz.

#### 2.5.2. Sensor-to-Sensor Rotation

Sensor orientations were calculated relative to adjacent sensors (e.g., left thigh to lumbar) using the SSRO approach. A KF [44,45] was used for the estimation, where, briefly, the direction of gravity and magnetic north in each sensor’s frame were assumed to be the same up to some varying rotation between the frames, expressed as

(8) g1=R21g2=q21⊗g2⊗q−121

(9) m1=R21m2=q21⊗m2⊗q−121,

where *g* is the direction of gravity (unit length vector), *m* is the measured magnetic field vector (can be raw or normalized), ⊗ indicates quaternion multiplication (where quaternion *q* multiplication of a vector *v* is expressed $q⊗v=q⊗\left[\begin{array}{cccc} 0 & v_{1} & v_{2} & v_{3} \end{array}\right]$), and q21 is the rotation quaternion (unit length) from frame 2 to frame 1. Time indices are left off for clarity unless different time points are used in the same equation. While magnetic field readings are frequently effected by magnetic disturbances, as the adjacent sensors are relatively close to each other, these disturbances were assumed to have an equivalent effect on both sensors. This in turn yields the assumption that the magnetic field vectors from both sensors were the same (albeit expressed in different frames).

The measured accelerometer signal (*a*) can be modeled as a linear combination of the true/body acceleration ($\tilde{a}$), gravitational acceleration, and white Gaussian measurement noise ($n_{a}$) as per

(10) $$\begin{array}{cc} a_{i} & =g_{i}+\tilde{a_{i}}+n_{a}. \end{array}$$

One component of the SSRO is then a model for estimating the direction of gravity. The state vector of the KF is defined as a combination of gravity vectors and a rotation quaternion between sensor 2 and sensor 1, per

(11) χ=g1Tg2TqT21T,

where χ is the state vector. The time update for this vector is defined using a first order approach, per

(12) $$\begin{array}{cc} s^{k} & =s^{k-1}+\Delta t{\dot{s}}^{k-1}, \end{array}$$

where *s* is a placeholder that can either be *g* or *q*. Because the gravity direction vector is part of the orientation matrix of its sensor, the same time derivative equation for matrices [46] can be used for the vector, per

(13) $$\begin{array}{cc} \dot{g_{i}} & =-\omega_{i}\times g_{i}=-\omega_{i}^{\times }g_{i}, \end{array}$$

where $\omega^{\times }$ is the skew symmetric matrix representation of ω. Similarly for the rotation quaternion, the time derivative can be related to the angular velocity [47] per

(14) $$\begin{array}{cc} \dot{q} & =-\frac{1}{2}q⊗\omega_{diff}=-\frac{1}{2}\omega_{diff}^{⊗}q, \end{array}$$

where $\omega_{diff}$ is the difference between angular velocities (i.e., ωdiff=ω2−RT21ω1) and $\omega^{⊗}$ is the 4×4 skew symmetric matrix

(15) $$\begin{array}{cc} q^{⊗} & =\left[\begin{array}{cccc} q_{0} & q_{x} & q_{y} & q_{z}\\ -q_{x} & q_{0} & -q_{z} & q_{y}\\ -q_{y} & q_{z} & q_{0} & -q_{x}\\ -q_{z} & -q_{y} & q_{x} & q_{0} \end{array}\right]. \end{array}$$

These time derivatives can then be substituted into the general time update equation (Equation (12)) as in

(16) $$\begin{array}{cc} g_{i}^{k} & =g_{i}^{k-1}-\Delta t\omega_{i}^{\times |k-1}g_{i}^{k-1}=\left(I_{3}-\Delta t\omega_{i}^{\times |k-1}\right)g_{i}^{k-1}=A_{g_{i}}g_{i}^{k-1} \end{array}$$

(17) $$\begin{array}{cc} q^{k} & =q^{k-1}-\frac{1}{2}\Delta t\omega_{diff}^{⊗|k-1}q^{k-1}=\left(I_{3}-\Delta t\omega_{diff}^{⊗|k-1}\right)q^{k-1}=A_{q}q^{k-1}, \end{array}$$

where $A_{g}$ and $A_{q}$ are the state update matrices for gravity and the rotation quaternion respectivelyand *I* is the identity matrix, with the subscript indicating the size. These matrices can be combined into one larger state update matrix for the state vector with $A_{g1}$, $A_{g2}$, and $A_{q}$ along the diagonal. By rearranging, the process covariance can be then be defined [34] as

(18) $$\begin{array}{cc} Q_{g_{i}} & =-\Delta t^{2}g_{i}^{\times }\left(\sigma_{\omega }^{2}I_{3}\right)g_{i}^{\times } \end{array}$$

(19) $$\begin{array}{cc} Q_{q} & =-\Delta t^{2}q^{⊗}\left(\sigma_{\omega }^{2}I_{4}\right)q^{⊗}, \end{array}$$

where $Q_{g}$ and $Q_{q}$ are the gravity and rotation quaternion process noise covariance matrices and $\sigma_{\omega }$ is the gyroscope measurement noise variance. These can be combined along the diagonal in the same way as the state update matrix.

In order to have a measurement for the gravity part of the KF, a first order model of the true acceleration was used [34], per

(20) $$\begin{array}{cc} {\tilde{a}}_{i}^{k} & =c{\tilde{a}}_{i}^{k-1}+\epsilon_{i}^{k}, \end{array}$$

where *c* is a constant between 0 and 1 which gives the cutoff frequency and ϵ is the time varying error of the model. Combined with Equation (10) and rearranged, an equality was formed:

(21) $$\begin{array}{cc} GI_{3}{\hat{g}}_{i}^{k} & =a_{i}^{k}-c{\tilde{a}}_{i}^{k-1} \end{array}$$

(22) $$\begin{array}{cc} H_{g}{\hat{g}}_{i}^{k} & =\zeta_{g_{i}}\equiv a_{i}^{k}-c{\tilde{a}}_{i}^{k-1} \end{array}$$

where ${\hat{g}}^{k}$ indicates the *a priori* estimate of $g^{k}$, *G* is the gravitational acceleration in $\frac{m}{s^{2}}$, *H* is the observation matrix, and ζ is the measurement. The measurement for the rotation quaternion was then a combination of the gravity vectors and magnetic field vectors. First, the rotation quaternion from $g_{2}$ to $g_{1}$ was found per

(23) q2∂(g^2,g^1)=cos12arccosg^2·g^1sin12arccosg^2·g^1g^2×g^1|g^2×g^1|,

where *∂* indicates a frame that is partially aligned with that of sensor 1 (i.e., rotation from gravity vectors does not account for heading). To complete the rotation to the frame of sensor 1, the magnetic field vectors can be used, first removing the component in the direction of gravity, per

(24) $$\begin{array}{cc} m_{xy|i} & =m_{i}-\left(m_{i}\cdot {\hat{g}}_{i}\right){\hat{g}}_{i}. \end{array}$$

The final rotation q∂1 can then be found from the rotation required to align q2∂⊗mxy|2⊗q−12∂ with $m_{xy|1}$, using the same method as for the gravity vectors. These partial rotations are then combined to yield the measurement per

(25) ζq=q∂1⊗q2∂.

Since measurement is directly provided for the rotation quaternion, the observation matrix for the rotation quaternion part of the state vector is simply $H_{q}=I_{4}$. As with the state update and process covariance matrix, the full observation matrix was formed with those of gravity and rotation quaternion on the diagonal. The gravity direction measurement noise covariance matrix was defined per [34]

(26) $$\begin{array}{cc} M_{g} & =\sigma_{a}^{2}I_{3}+\frac{c^{2}}{N}\sum_{j=1}^{N}{\tilde{a}}^{\left(k-j\right)}{\tilde{a}}^{{\left(k-j\right)}^{T}}, \end{array}$$

where *M* is the measurement noise covariance matrix, $\sigma_{a}$ is the accelerometer noise variance, and *N* is the number of samples used for the moving average. The rotation quaternion measurement noise covariance was defined as

(27) $$\begin{array}{cc} M_{q} & =\mu \left(\left|\right|{\tilde{a}}_{1}{\left|\right|}_{2}+\left|\right|{\tilde{a}}_{2}{\left|\right|}_{2}\right)I_{4}, \end{array}$$

where μ is an error factor term. Gravity direction and rotation quaternion matrices were combined as the other matrices. With the state update and measurement models, the rotation between two sensors could be obtained for the given MIMU data in the KF framework per

(28) $$\begin{array}{cc} {\hat{\chi }}^{k} & =A\chi^{k-1} \end{array}$$

(29) $$\begin{array}{cc} {\hat{P}}^{k} & =AP^{k-1}A^{T}+Q \end{array}$$

(30) $$\begin{array}{cc} K^{k} & ={\hat{P}}^{k}H^{T}{\left(H{\hat{P}}^{k}H^{T}+M\right)}^{-1} \end{array}$$

(31) $$\begin{array}{cc} x^{k} & ={\hat{x}}^{k}+K^{k}\left(\zeta^{k}-H{\hat{\chi }}^{k}\right) \end{array}$$

(32) $$\begin{array}{cc} P^{k} & =\left(I_{10}-K^{k}H\right){\hat{P}}^{k}, \end{array}$$

where *P* is the state covariance matrix, and *K* is the Kalman gain. Finally, at each time point, the true acceleration was calculated per

(33) $$\begin{array}{cc} {\tilde{a}}_{i}^{k} & =a_{i}^{k}-Gg_{i}^{k}, \end{array}$$

where $g_{i}$ comes from part of the state vector χ, depending on *i* (e.g., i=1 would use the first 3 elements, while i=2 would use the 3rd through 6th elements), and *G* is the gravitational acceleration in $\frac{m}{s^{2}}$.

This leaves five parameters to be set for the orientation estimation, $\sigma_{\omega }$, $\sigma_{a}$, *c*, *N*, and μ. $\sigma_{\omega }$ and $\sigma_{a}$ were determined to be $1\times 10^{-3}$ and $6\times 10^{-3}$ respectively for the Opal sensors—determined approximately from the sensors when placed on a stationary surface, while the *c* was set to 0.003 and *N* to 64. μ was set to $5\times 10^{-8}$ which balanced out the reliance on angular velocity integration for the rotation quaternion with using the gravity and magnetic field vectors.

Initialization of the gravity vectors for the KF used the mean of the first few samples of the measured acceleration, while the rotation quaternion was initialized by computing the measurement value in the KF.

#### 2.5.3. Sensor-to-Segment Alignment

The proposed method for estimating sensor-to-segment alignments utilizes the ability to estimate joint center locations to find the anatomical axes of the thigh and pelvis.

Joint center locations were estimated by leveraging inherent kinematic constraints as described previously [48,49]. Given two adjacent segments with sensors on each, that are linked via a common joint, the acceleration of the joint center as computed from each sensor’s measurements must be equal to within a relative rotation between frames. Mathematically, this is defined per

(34) a¯1k=a1k−ω1×ω1×+ω˙1×1kr1=Rk21a2k−ω2×ω2×+ω˙2×2kr2,

where $\bar{a}$ is the acceleration at the joint center and $r_{i}$ is the vector from the joint center to the ith sensor’s origin. With some manipulation, Equation (34) can be rearranged into an indeterminate linear system. The joint center location is fixed over time allowing the formation of the over-determined linear system, expressed as

(35) ω1×ω1×+ω˙1×11−R121ω2×ω2×+ω˙2×21⋮⋮ω1×ω1×+ω˙1×1N−RN21ω2×ω2×+ω˙2×2Nr1r2=a11−R121a21⋮a1N−RN21a2N,

where the superscripts indicate the time index. A minimum of 1500 observations were used to inform a least-squares solution for the joint center locations $r_{1}$ and $r_{2}$. Points were selected by considering the largest angular velocities measured by sensors 1 and 2.

While the hip experiences enough 3D rotation for good location estimation, the quasi-1D nature and limited AA and IER ranges of the knee can result in the estimated location lying anywhere along the knee FE axis. A correction for this location error relies upon an estimate of the knee FE axis found via enforcing the following kinematic constraint:

(36) $$\begin{array}{cc} \left|\right|\omega_{1}^{k}\times j_{1}{\left|\right|}_{2}-\left|\right|\omega_{2}^{k}\times j_{2}{\left|\right|}_{2} & =0, \end{array}$$

where $j_{i}$ is the rotation axis in the ith sensor’s reference frame. The rotation axes can be found via a least squares minimization algorithm such as gradient descent as in [14,15]. Once the axes have been obtained, the knee joint centers are corrected per

(37) $$\begin{array}{cc} r_{i} & ={\hat{r}}_{i}-j_{i}\frac{\left({\hat{r}}_{1}\cdot j_{1}\right)+\left({\hat{r}}_{2}\cdot j_{2}\right)}{2}, \end{array}$$

where ${\hat{r}}_{i}$ is the initial estimate of the knee joint center in the *i*-th sensors frame obtained from Equation (35). For the knee this would be the shank and thigh sensors. This process shifts the estimated joint center location close to the sensors, and results in a better approximation of the true joint center.

Once the joint centers were calculated, the pelvis and thigh fixed axes were calculated following the conventions for axes directions in [37]. The pelvis and thigh anatomical frames were then formed using a static standing trial as follows:

1. Rotate the fixed axes into common frames (ex. left thigh fixed axis from left thigh sensor frame to pelvis sensor frame).
2. Create the left and right hip joint coordinate systems as per ISB standards [37].
  $e_{1}$ = Pelvis fixed axis.
  $e_{3}$ = Left/right thigh fixed axis.
  $e_{2}$ = $e_{3}\times e_{1}$
3. Create the pelvis anatomical frame from the hip joint coordinate systems as:
  Z = $e_{1}$
  X = $\frac{1}{2}\left(e_{2,left}+e_{2,right}\right)$
  Y = z×x
4. Create the thigh anatomical frame from the hip joint coordinate system as:
  y = $e_{3}$
  x = $e_{2,left/right}$
  z = x×y

The anatomical frames and axes were created during the most still period of the static calibration trial, and remained constant relative to their respective sensors (e.g., left thigh anatomical frame is constant in left thigh sensor frame).

#### 2.5.4. Joint Angles

Following the sensor-to-segment alignments found in the proposed method, hip joint angles were then calculated by rotating the thigh anatomical frames into the pelvis frame at each time point using the SSRO algorithm described above, and computing the angles as per Equations (3)–(5).

All analysis was performed in Python 3.7, and the algorithms presented herein have been developed into a package, which can be found at https://github.com/M-SenseResearchGroup/pykinematics.

### 2.6. Validation

#### 2.6.1. Relative Orientations

Angles from the axis-angle convention for rotation were extracted from the sensor-to-sensor relative orientations from the thigh to lumbar sensor (i.e., R21 for the OMC and APDM methods and q21 for the SSRO method). SSRO and APDM angles were downsampled to 100 Hz to match those from OMC.

To ensure that RMSE values accurately reflected the minimum distance between angles, any angle differences larger than $180^{{}^{\circ}}$ had $360^{{}^{\circ}}$ either added or removed, depending on the sign of the difference (i.e., an angle difference of $350^{{}^{\circ}}$ would actually be $350-360=10^{{}^{\circ}}$). RMSE was then calculated per

(38) $$\begin{array}{cc} RMSE_{\alpha } & =\sqrt{\frac{1}{N}\sum_{k=1}^{N}\left(\Delta \alpha \right)}, \end{array}$$

where *N* is the number of samples in the trial and Δα is the corrected angle difference.

Additionally, regression analysis was performed between the SSRO and APDM methods and the OMC ground truth (e.g., OMC angles were the independent variable, and SSRO or APDM angles the dependent). This yielded slope and intercept values; slope giving an indication of how well changes in the SSRO or APDM rotation angles track those in the OMC rotation angles. Intercept provides a measure of the bias.

The mean and standard deviation (SD) of statistics were reported for each comparison (SSRO vs. OMC, APDM vs. OMC), trial type (star calibration and walking).

#### 2.6.2. Joint Angles

Agreements between FE, AA, and IER hip angles using the proposed method to those from OMC were established via RMSE, slope, intercept, ROM difference (ROMD), and drift. To provide context for these results, the same quantities were computed for the comparison between the FC-STI and OMC methods. Proposed and FC-STI method joint angle results were downsampled from 128 Hz to 100 Hz to match OMC joint angle results. RMSE is typically reported for assessing agreement for angle estimations, while slope and intercept provide information about tracking changes and bias respectively. Hip ROM is a clinically-relevant metric that has been shown to differ statistically significantly between, for example, persons with MS and healthy controls [2] and between those with PD and healthy controls [3]. As such, assessing the ROMD between the MIMU and OMC methods is an important component of establishing the applicability of the algorithm to clinical settings.

RMSE was computed the same way as for the orientation Euler angles, as were slope, and intercept. Slope values were taken as excellent if they were within 0.1 of 1, good from 0.3 to 0.1 away from 1, and moderate for values between 0.5 to 0.3 away. ROM values for each trial were computed from the difference between the maximum and minimum angles. If the trial was longer than 30 s however, then the first 10 s were excluded to ensure start-up effects were not present in the ROM estimates. ROMD was then the simple difference between proposed or FC-STI method ROMs and those from OMC.

Finally, the drift was assessed during the walking trials with linear regression analysis of the hip angle difference over time. Assessment of drift provides information regarding the ability of the KF framework to mitigate this error source during long non-stationary tasks, which is a critical aspect of most MIMU joint angle estimation algorithms. We do not assess drift in the star calibration trials as other sources of error (e.g., axis misalignment, bias) dominate, which causes the linear model-based approach to make unreliable estimates of the drift error. Drift distributions for each method were tested using the Wilcoxon test for non-zero slopes.

The mean and standard deviation (SD) of statistics were reported for each comparison (proposed vs. OMC, FC-STI vs. OMC), trial type (star calibration and walking), and angle (FE, AA, IER).

## 3. Results

### 3.1. Orientation

Table 1 reports the agreement of methods for computing orientation of the lumbar sensor relative to the left and right thigh sensors with OMC. The SSRO method exhibits much lower mean RMSE values ($11.82^{{}^{\circ}}$ and $12.32^{{}^{\circ}}$ for star calibration and walking) than the APDM orientation ($21.61^{{}^{\circ}}$ and $23.76^{{}^{\circ}}$). Slopes for all trials and methods were above 0.85, indicating that both methods tracked the ground truth OMC angle with less than 15% variation. Intercept values for the SSRO method were again better than APDM, though for both methods very large SDs were present indicating substantial variability in results between subjects.

### 3.2. Joint Angles

**Figure 2:**
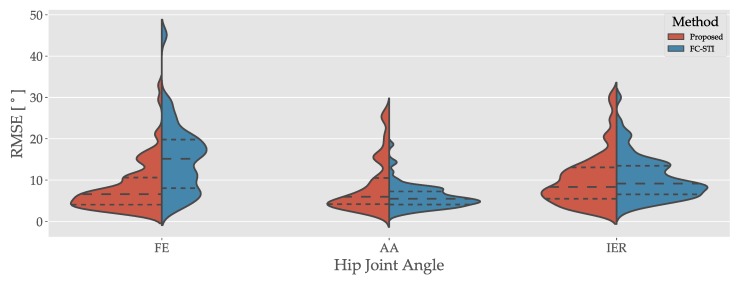
Root mean square error (RMSE) values for all angles and trials from the proposed and functional calibration—strap down integration (FC-STI) methods. Red is for the proposed method results, blue for the FC-STI method results. FE: flexion/extension, AA: ad/abduction, IER: internal-external rotation.

The violin plots of Figure 2 show the hip angle RMSE in each anatomical direction for the proposed and FC-STI methods. Results demonstrate that the median FE RMSE for the proposed method is below that of the FC-STI method. Inter-quartile ranges (IQRs) for AA and IER angles from both methods are overlapping, indicating similarity between the results.

**Figure 3:**
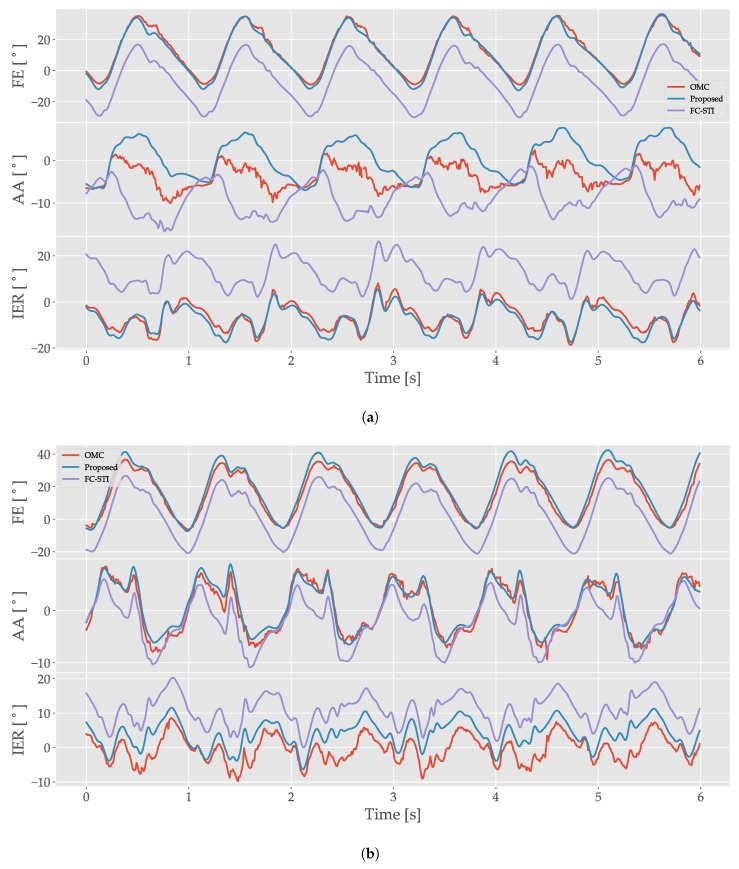
Sample gait cycles for the right hip from example subjects who exhibited (**a**) good FE and IER angle tracking, though with larger offsets for the FC-STI method and (**b**) good angle tracking for all three angles. Data were sampled between 20 th and 26 th seconds of the 60s trial. For both subfigures: (**top**) FE (flexion/extension) angle traces. (**middle**) AA (ad/abduction) angle traces. (**bottom**) IER (internal-external rotation) angle traces. Red is the OMC trace, blue is the proposed method trace, purple is the FC-STI trace.

Figure 3 shows several sample cycles of gait during fast walking on a treadmill for two different subjects. For both subjects, FE and IER proposed method angles exhibit close agreement with the OMC estimate. While the FC-STI method results track these angles well, there is a larger offset present. For the AA angles, the two subjects differ in tracking performance. The subject in Figure 3a shows poor tracking for both methods, while the subject in Figure 3b shows good tracking. Collectively, these results suggest that the proposed approach improves FE angle estimation while maintaining performance in the other anatomical directions.

**Figure 4:**
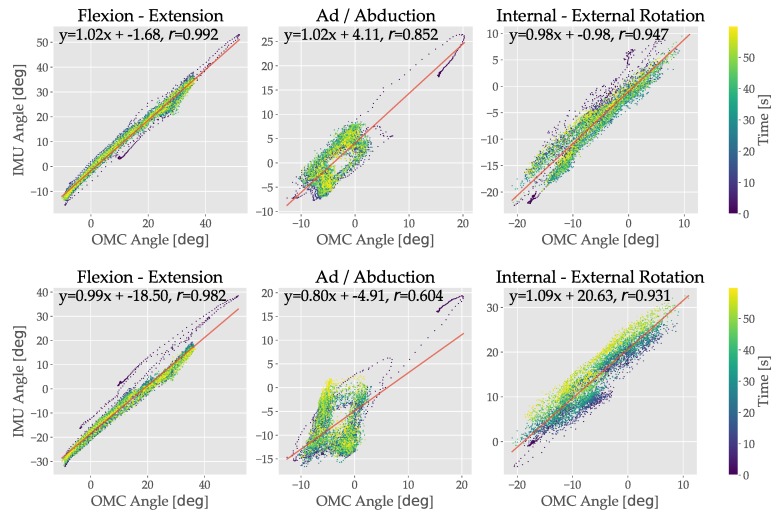
Sample regression plots for the treadmill fast walking trial for the right hip, comparing the proposed and FC-STI to OMC methods. The same trial and subject as shown in Figure 3a is shown. (**top**) Regressions for the proposed method, with FE, AA, and IER plotted from left to right. (**bottom**) Regressions for the FC-STI method, with FE, A, and IER plotted from left to right.

These results for the subject in Figure 3a are reflected in the sample regression plots from the treadmill fast walk trial of Figure 4. Good agreement is observed between the proposed method and OMC, with slopes and *r* values that are close to 1. The circular pattern in the AA graphs is due to the cyclic nature of the motion, with proposed and FC-STI results cycling away and towards the OMC ground truth during each gait cycle. This is reflected in the lower *r* values, 0.852 and 0.604 for the proposed and FC-STI AA angles respectively.

The full report of results is found in Table 2. For the star calibration, the proposed method had half the FE RMSE ($7.88\pm 3.64^{{}^{\circ}}$) compared to the FC-STI method ($14.49\pm 6.28^{{}^{\circ}}$). Proposed AA and IER RMSE were higher than those of the FC-STI method, but still within the 1 SD range. Slopes were comparable for all three angles between both methods, as were intercepts, though FE and IER for the proposed method were slightly lower than the FC-STI method values. For the star calibration, the proposed method ROMD values were higher than those of the FC-STI method, though both methods showed large SDs.

Walking results showed similar trends to those in the star calibration. FE RMSE for the proposed method was approximately half that of the FC-STI method ($8.62\pm 7.52^{{}^{\circ}}$ compared to $15.64\pm 10.24^{{}^{\circ}}$), AA was higher ($5.03\pm 6.42^{{}^{\circ}}$ compared to $5.65\pm 3.16^{{}^{\circ}}$), and IER was slightly lower ($9.99\pm 5.90^{{}^{\circ}}$ compared to $11.93\pm 6.04^{{}^{\circ}}$). Slopes were again consistent across both methods, indicating that both methods do track the ground truth OMC angles well. Intercepts followed a similar trend to that of the RMSE values, with FE being lower for the proposed method ($-6.29\pm 9.15^{{}^{\circ}}$ compared to $-10.17\pm 14.75^{{}^{\circ}}$) and AA and IER being similar. ROMD values were very small for both methods, under $2.2^{{}^{\circ}}$ for the proposed and under $4.5^{{}^{\circ}}$ for the FC-STI method. SD values were also much smaller than those of the star calibration, indicating a tight clustering of ROMD values around zero. For ROMD, FE values from the proposed and FC-STI methods were similar, while the proposed method AA ($0.80\pm 4.64^{{}^{\circ}}$) and IER (0.77±4.34) were much lower than those of the FC-STI method ($3.35\pm 5.55^{{}^{\circ}}$ and $4.49\pm 6.78^{{}^{\circ}}$ respectively). Wilcoxon *p*-values showed statistical evidence that AA and IER drift values for both the proposed and FC-STI methods were not zero, especially FC-STI IER, with a drift of $-0.12\pm 0.18^{{}^{\circ}}$/s.

## 4. Discussion

In this study, novel sensor-to-sensor relative orientation and 3D joint angle estimation methods were presented. Only minimal calibration motions are required, and no specific sensor orientation or placement is necessary. Validation was performed on human subjects, using an OMC system as ground truth, with performance relative to existing methods established by reporting existing algorithm performance.

The proposed orientation method (SSRO) strongly outperforms the existing proprietary APDM orientation, with SSRO RMSE values approximately half those of the APDM (Table 1) for both the star calibration and treadmill fast walking. SSRO slopes were slightly closer to 1 than the APDM slopes, though large SD ranges indicate little difference between the two methods. Overall, the SSRO shows excellent tracking of the ground truth OMC orientations, though with some offset that is reflected in the higher RMSE and intercept values. Better performance of the SSRO method is likely due to better compensation for magnetic effects under the assumption that both sensors pick up the effect. Additionally, there are likely performance benefits from direct alignment of gravity and magnetic field vectors between sensors.

Proposed method RMSE values were all below $10.5^{{}^{\circ}}$ when compared to OMC for both trials, and slopes were all excellent (0.90–1.04) except for IER during walking, which was good (0.78). The same comparison for the FC-STI method yielded similar RMSEs for AA and IER directions, though proposed FE RMSEs were just over half of those from the FC-STI method (Table 2), as well as similar slopes. As the FC-STI method was originally designed and utilized for alpine skiing, this study also serves as a validation during the more common daily activity of gait. The original study for the FC-STI method reported mean errors of $-10.7\pm 3.4^{{}^{\circ}}$, −3.3±4.1, and $0.5\pm 4.8^{{}^{\circ}}$ for FE, AA, and IER angles [11]. Here, we observed mean errors of $6.35^{{}^{\circ}}$, $3.81^{{}^{\circ}}$, and $6.24^{{}^{\circ}}$ for proposed method FE, AA, and IER in the fast treadmill walk, and $10.71^{{}^{\circ}}$, $1.84^{{}^{\circ}}$, and $-5.50^{{}^{\circ}}$ for the FC-STI method, all of which are comparable or better (e.g., proposed FE) with the original study [11]. The performance of the proposed method for FE angles over the FC-STI method is likely due to the definition of the rotation axes. Whereas the proposed method uses a definition closely aligned with that of OMC, the FC-STI method relies on calibration motions that are assumed to align with the joint rotation axes and may result in more cross-talk between axes and therefore higher RMSE values, especially for FE which is the prominent axis of motion.

Few other studies have implemented or validated hip angle algorithms in human subjects. Validation of a proprietary algorithm during manual material handling tasks reported less than $7.5^{{}^{\circ}}$ RMSE [31]. Another proprietary algorithm validation resulted in $9.6^{{}^{\circ}}$ and $27.6^{{}^{\circ}}$ RMSE for walking and running on a treadmill [35]. While these results are comparable or worse, no information regarding the algorithms employed by the sensor systems was given due to their proprietary nature, and the significant performance decrease from walking to running raises concerns regarding the utility of the systems. Additionally, the use of alternative sensors would not be possible with the proprietary nature of the algorithms. During walking, another study, which did not use proprietary algorithms, reported mean errors less than $6^{{}^{\circ}}$ [32]. However, upon detection of significant drift, trials were removed from analysis, resulting in approximately 60% of trials being removed (57% in the IER direction). Additionally, angles were taken to be $0^{{}^{\circ}}$ during static standing, which may not be repeatable, or valid in populations with balance, mobility, or joint ROM impairments.

Drift in the joint angles was assessed for the proposed and FC-STI methods, with similar results. Both AA and IER showed statistical evidence of non-zero drift during walking for both methods. All drift value mean magnitudes were less than 0.03${}^{{}^{\circ}}$/s except walking FC-STI IER ($-0.12^{{}^{\circ}}$/s). We suspect that there is evidence of drift in the walking trial (significant non-zero slopes in the AA and IER directions) because there are no still periods. For the SSRO KF, this impacts estimates of the direction of gravity. The treadmill walking task was one minute long, and therefore future work should explore this trend over multiple minute-long tasks with the goal of developing effective mitigation strategies.

Joint ROM is a highly relevant metric for a variety of clinical populations [1,2,3]. As such, assessing the difference between ROM from OMC and wearable sensor methods is critical to establishing performance. While the proposed method exhibits large ROMD with OMC during the star calibration task ($8.74^{{}^{\circ}}$ to $18.08^{{}^{\circ}}$), this set of motions is not typically performed. Gait ROMD yields a much more informative assessment for clinical application, and the proposed method shows very strong performance, with $2.17^{{}^{\circ}}$, $0.80^{{}^{\circ}}$, and $0.77^{{}^{\circ}}$ mean ROMD for FE, AA, and IER respectively. The FC-STI method also performs very well, just slightly higher at $1.86^{{}^{\circ}}$, $3.55^{{}^{\circ}}$, and $4.49^{{}^{\circ}}$ mean ROMD.

Differences in ROM between healthy and impaired populations indicate that the levels of results achieved especially by the proposed method are sufficient to detect reported ROM differences. For example, in persons with OA, mean peak hip extension in the stance phase during walking was reported as $8.4\pm 7.0^{{}^{\circ}}$ compared to $14.2\pm 8.7^{{}^{\circ}}$ for healthy controls [1], a $5.8^{{}^{\circ}}$ difference in means. In another study on persons with MS, hip ROM during stance was reported as $35.58\pm 4.91^{{}^{\circ}}$ during walking compared to $39.26\pm 3.84^{{}^{\circ}}$ for healthy controls [2], a $3.68^{{}^{\circ}}$ difference in mean ROM. People afflicted by PD showed the biggest difference to healthy control, with sagittal plane (similar to FE) ROM values of $47.6\pm 4.1^{{}^{\circ}}$, $33.2\pm 8.5^{{}^{\circ}}$, and $41.5\pm 5.1^{{}^{\circ}}$ for healthy controls, PD patients off Levadopa (PD treatment drug), and PD patients on Levadopa respectively [3]. For the proposed method, FE ROMD was $2.17\pm 3.61^{{}^{\circ}}$ during treadmill walking, indicating that the proposed method has the resolution necessary to observe these differences between healthy and afflicted populations. With that said, future work should explore similar validation studies to establish performance characteristics of the proposed approach in these populations.

Future work on the SSRO should involve an observability and stability analysis of the KF form [50] to assess the ability of the KF to capture the dynamics of the problem. Future work should also involve a comparison against fluoroscopic methods in an effort to provide an even more detailed picture of the performance of the proposed approach. To this end, future work should also examine the repeatability of the proposed method to provide evidence for its use in studies looking for changes in kinematics over repeated visits. Additionally, while previous studies have not grouped by gender [31,32,35], this should be explored in the future to examine potential performance changes associated with anatomical differences. Finally, extension and modification of the proposed methods to other joints (e.g., the knee, shoulder, etc.) should be explored.

Limitations of this study include the limited age range of subjects, with little to no representation of subjects over the age of 25, as well as the small subject pool. While typical for similar studies (N = 11 in [11]) a larger subject pool would allow for stronger conclusions to be drawn from the results. Furthermore, the testing on healthy subjects does not indicate how well the algorithms will perform on individuals with mobility impairments. Additionally, while effort was made to ensure that subjects were instrumented for OMC by the same person, in the same way, marker placement was done by two people, potentially introducing some variability between subjects in the initial creation of anatomical reference frames. Finally, while the sensor assigned to the pelvis is placed in the manufacturer’s recommended location, which is also used in previous studies [11,32], it is not directly on the pelvis which could result in some rotations from the spine affecting results.

## 5. Conclusions

Wearable MIMU-based methods for estimating sensor-to-sensor relative orientation and 3D hip joint angles were proposed and validated against OMC on human subjects. Python implementations of these algorithms have been made available as open-source software. Innovations in sensor-to-sensor orientation and sensor-to-segment alignment yield improvements in estimation performance during the walking and star calibration tasks considered herein. Specifically, the SSRO showed much lower RMSE values (12.32 vs. $24.61^{{}^{\circ}}$) than the proprietary orientation estimation algorithm provided by a commercially available MIMU system. Similarly, the proposed method for estimating hip joint angles also had lower RMSE (8.62 vs. $15.64^{{}^{\circ}}$) than the FC-STI method. During walking, ROMDs for the proposed method were all below $2.17^{{}^{\circ}}$, further indicating their close agreement with OMC and the potential clinical utility of this approach. Overall, these results are comparable to and improve upon existing methods for estimating hip joint angles with wearable sensors.

## Figures and Tables

**Table 1:** Mean (SD) of statistics for agreement between sensor-to-sensor relative orientations (SSRO) and APDM results and ground truth optical motion capture (OMC) rotation angles from the axis-angle representation.

Trial	Method	RMSE (${}^{{}^{\circ}}$)	Slope	Intercept (${}^{{}^{\circ}}$)
Star Calibration	SSRO	12.32 (13.89)	0.91 (0.18)	14.51 (30.07)
	APDM	24.61 (18.59)	0.85 (0.51)	21.91 (71.24)
Walking	SSRO	11.82 (11.53)	0.95 (0.17)	8.10 (26.81)
	APDM	23.76 (21.61)	0.85 (0.51)	22.07 (63.78)

**Table 2:** Mean (SD) of several statistics comparing the proposed and FC-STI results to those of OMC. Left and right hip angles are combined for the star calibration and treadmill fast walk results. Bold text for drift indicates Wilcoxon p<0.05 with the null hypothesis that the drift is 0.

Trial	Method	Angle	RMSE (${}^{{}^{\circ}}$)	Slope	Intercept (${}^{{}^{\circ}}$)	ROMD (${}^{{}^{\circ}}$)	Drift (${}^{{}^{\circ}}$/s)
Star Calibration	Proposed	FE	7.88 (3.64)	1.04 (0.09)	−2.91 (6.48)	18.08 (12.57)	-
		AA	9.16 (6.69)	0.98 (0.21)	−5.45 (7.45)	8.74 (14.93)	-
		IER	10.36 (7.04)	0.90 (0.56)	−2.53 (9.79)	10.21 (14.33)	-
	FC-STI	FE	14.49 (6.28)	1.04 (0.07)	−4.48 (14.20)	16.35 (12.38)	-
		AA	6.24 (2.61)	0.96 (0.18)	−2.21 (4.22)	3.13 (10.34)	-
		IER	8.96 (3.90)	0.95 (0.33)	−4.12 (7.46)	4.58 (7.69)	-
Walking	Proposed	FE	8.62 (7.52)	1.00 (0.07)	−6.29 (9.15)	2.17 (3.61)	−0.00 (0.03)
		AA	8.03 (6.42)	0.94 (0.12)	−3.93 (9.00)	0.80 (4.64)	**0.01 (0.02)**
		IER	9.99 (5.90)	0.78 (0.31)	−5.92 (8.69)	0.77 (4.34)	**−0.03 (0.07)**
	FC-STI	FE	15.64 (10.24)	0.97 (0.06)	−10.17 (14.75)	1.86 (3.44)	−0.01 (0.03)
		AA	5.65 (3.16)	0.95 (0.12)	−1.97 (5.07)	3.35 (5.55)	**0.03 (0.06)**
		IER	11.93 (6.04)	0.76 (0.22)	6.19 (1.28)	4.49 (6.78)	**−0.12 (0.18)**
